# Management of Neuraxial Analgesia in a Parturient with Factor XIII Deficiency: A Case Report and Proposed Management Algorithm

**DOI:** 10.1155/2020/8892225

**Published:** 2020-12-31

**Authors:** David B. Carroll, Conrad Myler, Natthapol Songdej, Khaled Sedeek, Dmitri Bezinover

**Affiliations:** ^1^Department of Anesthesiology and Perioperative Medicine, Milton S. Hershey Medical Canter, 500 University Dr, Hershey, PA 17033, USA; ^2^Department of Medicine, Division of Hematology and Oncology, Milton S. Hershey Medical Center, 500 University Dr, Hershey, PA 17033, USA

## Abstract

Factor XIII (FXIII) deficiency is a rare coagulation defect that can be associated with significant bleeding. A 28-year-old pregnant woman, with a history of hemorrhagic stroke secondary to severe congenital FXIII deficiency, presented in active labor requesting an epidural. Factor XIII levels had been monitored throughout her pregnancy and treated with intermittent factor XIII infusions to maintain factor levels above 30% of normal. After careful multidisciplinary peripartum evaluation and FXIII replacement, neuraxial analgesia was performed without complication. Neuraxial analgesia can be performed without complication in patients with FXIII deficiency if FXIII levels are carefully managed and no other coagulopathy exists.

## 1. Introduction

Factor XIII (FXIII) deficiency is a rare inherited autosomal recessive condition with an incidence of 1 in 2 million [1] and is associated with easy bruising, impaired wound healing, persistent umbilical cord bleeding, and recurrent fetal loss [2]. The first report describing an association between FXIII deficiency and significant bleeding which resolved after fresh frozen plasma administration was published in 1960 [3]. FXIII, also called fibrin stabilizing factor, accelerates fibrin crosslinking and plays an important role in preventing clot lysis. FXIII also plays a role in uterine hemostasis and placental maintenance. Pregnant patients with FXIII deficiency are at increased risk of placental abruption and hemorrhage.

There are currently no recommendations pertaining to the placement of neuraxial analgesia or anesthesia in pregnant patients with FXIII deficiency. This report describes successful epidural catheter placement for labor analgesia in a patient with known severe FXIII deficiency. In addition, we propose an algorithm for performing neuraxial analgesia in patients with FXIII deficiency. The patient gave HIPAA authorization for publication of this manuscript, which adheres to the applicable EQUATOR guidelines.

## 2. Case Presentation

A 28-year-old, 60 kg patient, ASA physical status III, G2P1001, at 39 2/7 weeks estimated gestational age (EGA) presented in active labor. The patient was told that at the time of her birth, significant umbilical cord bleeding was encountered, but no testing was performed. FXIII deficiency was diagnosed when the patient was two years old after a minor fall resulted in a subdural hemorrhage requiring decompressive craniotomy. Coagulation studies at that time confirmed a congenital FXIII deficiency with a baseline FXIII level of 5%. Prior to this pregnancy, she had been on infusions of 45 U/kg of Factor XIII Concentrate (Corifact, CSL Behring) every four weeks.

Her previous successful pregnancy was managed with FXIII replacement, with intravenous remifentanil patient-controlled analgesia (PCA) for analgesia at delivery. She described the delivery as “painful” and “traumatic.”

During the present pregnancy, FXIII was initially maintained above 10% with an infusion of 30 U/kg of FXIII concentrate every two weeks from week 10 until week 23 of her pregnancy and then 50 U/kg every two weeks until week 37. After 37 weeks, she received an infusion of 50 U/kg every week.

She presented in active labor at term, one day after FXIII administration, requesting neuraxial analgesia. Her review of systems and physical exam did not reveal any signs of bleeding. Her hemoglobin was 12.5 g/dL and platelet count 252 × 10^9^/L, and factor XIII activity level was estimated by the hematologist to be greater than 50% of normal with FXIII administered less than 24 hours prior and based on her previous response. Quantitative FXIII testing was pending. After discussing the risks, benefits, and alternatives including unmedicated delivery and another remifentanil PCA, the patient refused consent for IV narcotics. She requested and provided informed consent for neuraxial analgesia. A 19G multiport epidural catheter was placed at the L3-L4 interspace without difficulty. A bolus of 5 mL of bupivacaine (1.25 mg/mL) and fentanyl (2 mcg/mL) was administered, and patient-controlled epidural analgesia was established at a basal rate of 8 mL/hour with a bolus dose of 3 mL available every 15 minutes. Satisfactory analgesia was maintained throughout labor, and approximately 2 hours later, the patient delivered a healthy baby girl (APGAR 8/9, weight 2860 grams). The catheter was removed soon after delivery with the tip intact. There were no signs of neurological deficits or symptoms of postpartum or epidural bleeding, and she was discharged home the next day.

## 3. Discussion

In this case, we describe the successful placement of an epidural catheter in a patient with severe FXIII deficiency.

FXIII plays a critical role in both stabilizing the fibrin clot and suppressing fibrinolysis via inhibiting the conversion of plasminogen to plasmin. FXIII is a heterotetramer composed of two A subunits, which act as catalysts, and two B subunits, which act as carriers. Thrombin cleaves a peptide bond off both A subunits. This leads to dissociation of both B units from the tetramer and conversion of FXIII to activated FXIII (FXIIIa) with subsequent fibrin crosslinking and enhanced clot stabilization [4, 5].

FXIII deficiency can either be congenital or acquired. Congenital deficiencies result from more than 70 different genomic mutations. The majority, and those with the most severe bleeding, are associated with mutations of subunit A (type 2). Less than 5% of clinically significant hemorrhage is related to mutations of subunit B (type 1) [5]. Acquired causes of FXIII deficiency result from the development of inhibitors binding directly to FXIII, seen with some autoimmune conditions, severe liver disease, inflammatory bowel disease, and as a side effect of the administration of some medications such as penicillin, phenytoin, and isoniazid [6]. Patients with an acquired deficiency have an FXIII activity of less than 50% and can present with spontaneous hematoma formation, delayed postoperative bleeding, or intracranial hemorrhage [7, 8].

Early signs of FXIII deficiency include umbilical cord bleeding, present in up to 80% of patients, intracranial bleeding in up to 30% (both of which our patient had), hematoma formation, and poor wound healing. Women have a significantly elevated risk of recurrent spontaneous miscarriages due to the risk of placental detachment [4, 5, 9].

Pregnant patients with FXIII deficiency should be managed by a multidisciplinary team that includes obstetrics, hematology, and anesthesiology. Antepartum consultation with an anesthesiologist, as occurred with this patient at 35 weeks EGA, should be offered to facilitate anesthetic planning. Having this consultation ahead of time is helpful to fully discuss the risks, benefits, and alternatives of various anesthetic approaches in obtaining informed consent. Intravenous analgesics including remifentanil PCA and inhaled nitrous oxide may be offered as alternatives to epidural analgesia.

Successful pregnancies in women with FXIII deficiency have been achieved with regular administration of plasma, cryoprecipitate, or FXIII concentrate. It is suggested that FXIII levels during pregnancy be maintained above 10% to prevent subchorionic hematoma formation which increases the risk of miscarriage [4]. At the time of labor and delivery, FXIII levels should be increased to above 20–30% to prevent placental abruption and postpartum hemorrhage [4]. Coagulation assessment can be challenging in these patients. Both quantitative and qualitative FXIII evaluations are necessary. A negative *qualitative* test (results of which can be obtained in a few hours) indicates that the patient does not have a critical FXIII deficiency (a level below 5–10% of normal). A *quantitative* test demonstrates the precise level of FXIII, but results may take up to 7 days to be available. In our patient, quantitative testing was performed on the day of delivery, and results were returned approximately one week later, demonstrating FXIII levels above 130% of normal.

Traditional coagulation studies (prothrombin time (PT)), international normalized ratio (INR), and partial thromboplastin time ((PTT) and bleeding time) are typically normal because these tests assess the early stages of the coagulation cascade before FXIII becomes involved [1]. A clot solubility test (CST) can be used (after exclusion of hypofibrinogenemia and dysfibrinogenemia), but the sensitivity and specificity are low [10]. Viscoelastic testing (VET) (maximal amplitude and lysis 60) is more sensitive to low levels of FXIII than the CST (10) and may help with decision-making regarding performing neuraxial analgesia [5, 11]. Other coagulopathy, such as thrombocytopenia, may preclude placement of neuraxial blocks.

FXIII deficiency management during pregnancy is complicated by the fact that not only does FXIII activity decrease during the second and third trimester, but the half-life of FXIII concentrate is also reduced (from approximately 7 days in nonpregnant patients to 1.8 days during the third trimester) [4]. One commonly used dosing regimen for FXIII replacement during pregnancy is 250 units (U) weekly from early in pregnancy until 23 weeks of gestation, followed by 500 U weekly thereafter, with a “booster” dose of 1000 U given during labor and delivery [4]. Others have recommended 10 U/kg every 1-2 weeks throughout pregnancy [2, 12]. Bleeding is rare at FXIII levels as low as 30% [13, 14]. Despite this, normal levels are considered to be 50% or above. Maintaining factor levels above 50% during the period of catheter insertion to 12–24 hours after catheter removal has been recommended in patients with hemophilia A (factor VIII deficiency), hemophilia B (factor IX deficiency), and type 1 von Willebrand disease [15]. For an additional measure of safety, an additional dose of FXIII (500–1000 U) should be administered on the day of a neuraxial procedure. This is necessary because of the decreased half-life of FXIII late in pregnancy and the lag time in obtaining FXIII level results. Although intrathecal injection with a small gauge spinal needle is generally considered to be lower risk for epidural hematoma than epidural catheter placement, both techniques may be performed without increased risk complication if FXIII is managed appropriately. If a neuraxial procedure is planned, and the patient has no other contraindications to neuraxial analgesia, but the level of FXIII is unknown, prudence would dictate that FXIII concentrate should be administered prior to the procedure. VET can help to exclude residual coagulopathy.

**Figure 1:**
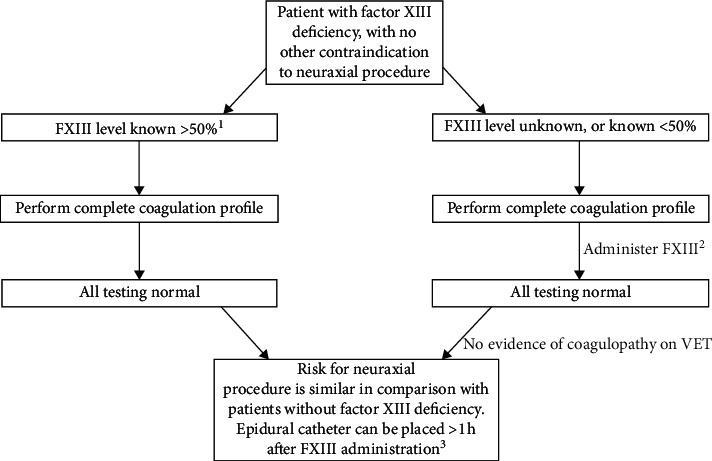
Algorithm for performing neuraxial procedures in patients with FXIII deficiency. VET: viscoelastic testing. ^1^FXIII activity may be known by quantitative testing on admission, or calculated by a hematologist, taking into consideration patient's medical conditions, comorbidities, timing of last dose, and quantitative assessment of prior response during the 3^rd^ trimester. ^2^Administration of FXIII may be necessary because of the time required to obtain results of a quantitative FXIII test. FXIII dosing should be based on the recommendation of the hematologist, targeting level above 50%. ^3^FXIII also confirmed or calculated above 50% for epidural catheter removal.

We have demonstrated that neuraxial anesthesia can be performed without complication in an obstetric patient with a history of severe FXIII deficiency. Before performing any neuraxial procedure, the patient's entire coagulation profile should be evaluated. The proposed algorithm (Figure 1) can be used for performing neuraxial anesthesia in any patient with FXIII deficiency though FXIII pharmacodynamics will differ in nonpregnant patients.

## Data Availability

The data used to support the findings of this study are included within the article.
